# Green Microbiology for Planetary Resilience: A Research Agenda for Microbial Sustainability Science

**DOI:** 10.1111/1758-2229.70444

**Published:** 2026-10-18

**Authors:** Adenike A. Akinsemolu, Helen Onyeaka

**Affiliations:** ^1^ The Green Institute Ondo Nigeria; ^2^ School of Chemical Engineering University of Birmingham Birmingham UK; ^3^ Birmingham Institute for Sustainability and Climate Action Birmingham UK

**Keywords:** circular bioeconomy, climate microbiology, environmental justice, green microbiology, microbial sustainability, one health, planetary resilience

## Abstract

The planet is facing a crisis occasioned by climate change, biodiversity loss, physical, chemical, and biological soil degradation, water insecurity and pollution, infectious disease crises, and unsustainable production systems and vulnerable food systems. These problems are deeply interconnected and share a common solution: microbial science. Microbes are central to maintaining or restoring water safety and quality, the carbon and nitrogen cycles responsible for maintaining and improving soil quality and fertility, plant productivity and immunity from pests and diseases, animal and human health and immunity, and other essential ecosystem services that maintain plant, animal, human, and environmental health. Despite the transformative potential of microbes and a growing body of recent scholarship recognizing their centrality to sustainability at a global scale, there is no coordinated, actionable research and policy agenda mobilizing microbial science toward planetary resilience. To this end, this perspective builds on established principles of Green Microbiology to advance a 10‐year, forward‐looking research agenda spanning climate science, food systems, water security, circular bioeconomy, One Health, biodiversity conservation, and artificial intelligence and their alignment to microbial research, technology, trends, and governance and policy structures with the fundamentals of planetary resilience.

## Graphical abstract

Green Microbiology integrates microbial science with sustainability to address interconnected global challenges. This framework outlines a 10‐year research agenda that positions microbes as central drivers of climate stability, food security, water quality, biodiversity conservation, and One Health to achieve long‐term planetary resilience.

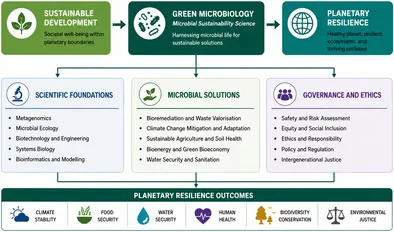

## 1 Chapter 1: Introduction

The twenty‐first century is facing a monumental crisis characterized by accelerating environmental and public health crises. Atmospheric temperatures are rising globally, extreme weather events are more frequent, various species are declining at previously unprecedented rates, aquatic and terrestrial ecosystems are losing biodiversity rapidly, agricultural soils are experiencing physical, chemical, and biological degradation, freshwater systems are heavily polluted and overexploited, environmental pollution is rising, and infectious disease outbreaks are on the rise all over the world (Pfenning‐Butterworth et al. 2024; Shivanna 2022; Ihsan et al. 2024). These challenges converge into a triple planetary crisis, which is estimated to cause millions of premature deaths globally every year from Antimicrobial Resistance (AMR), cardiac, respiratory, and infectious diseases, food shortages, and malnutrition (Ihsan et al. 2024; Fuller et al. 2022).

Deforestation and the continued use of fossil fuels release greenhouse gases that trap heat in the atmosphere, raising global temperatures. The rising temperatures modify marine and freshwater ecosystems and melt glaciers, leading to rising water levels that displace coastal communities (Shivanna 2022). The resulting collapse of marine ecosystems on which fish depend reduces the food supply for millions who depend on fishing as a source of food and income. On land, heatwaves and droughts reduce agricultural yield, deepening food insecurity, particularly among the world's most vulnerable populations. Rising temperatures are also changing the timing of seasons and leading to misaligned animal and insect migration timelines, causing plants to flower before their pollinators arrive (Shivanna 2022). This reduces the yield of food crops while disrupting reproduction in wild plant species, further weakening ecosystems. Meanwhile, air pollution from burning fuels, agricultural chemicals, and greenhouse gases is leading to respiratory disease and compromising immune systems amid a worsening infectious disease crisis as emerging and re‐emerging infectious diseases, often with environmental and zoonotic origins, destabilize public health systems worldwide (Pfenning‐Butterworth et al. 2024; Shivanna 2022).

Microorganisms sit at the intersection of these planetary crises. Microbes are central to Earth's system processes. Microbial activity drives the carbon cycle, the nitrogen cycle, the phosphorus cycle, the hydrological cycle, and the oxygen cycle. Soil fertility is a product of the complex interactions between bacteria, fungi, archaea, protozoa, plant roots, and organic matter, with soil microbiota playing crucial roles in its composition, structure, and quality (Wang et al. 2024). Microbial communities are equally important in the development of the immune systems of human beings and animals and the production of oxygen, with a significant percentage of the planet's oxygen being produced as a byproduct of photosynthesis in microorganisms such as cyanobacteria (West 2022). The decomposition of waste, mineralization of nutrients, and cycling of crucial minerals such as iron are all mediated by microorganisms as well, making the biosphere heavily reliant on microorganisms for maintenance, survival, and regeneration.

Traditional microbiology recognizes the important roles of microorganisms and has generated extensive knowledge and technologies leveraging their unique abilities, driving transformative inventions and applications in medicine, agriculture, industry, and biotechnology. However, the field has not been integrated sufficiently into sustainability science. In recent years, a growing body of scholarship has begun to articulate the potential of microorganisms to contribute to climate change mitigation, circular resource management, ecosystem maintenance and restoration, food security, water treatment and remediation, and overall planetary health, with recent calls to action arguing that microorganisms are essential to achieving the UN Sustainable Development Goals (Crowther et al. 2024), that they are conspicuously absent from global sustainability frameworks despite their centrality to climate stability, food security, and biodiversity (Webster 2026), and that the microbiosphere itself may serve as an early‐warning system for planetary change (Baquero et al. 2026). The International Microbiology Literacy Initiative (IMiLI) champions public and policy understanding of microbial processes as essential to sustainable decision‐making and has built a global education infrastructure toward that end (Timmis et al. 2019). However, a unifying scientific framework that aligns microbial science with the full spectrum of sustainability challenges is yet to be established.

This Perspective builds on earlier principles of Green Microbiology as outlined by Akinsemolu (2023), articulating them in relation to sustainable development, to advance a research agenda for microbial sustainability science and planetary resilience. The framework is premised on the need for microbial science to move beyond observation and application toward active solutions for the planetary crisis. It calls for a new generation of microbiologists who understand and leverage their science not only as a technical discipline but as a contributor to the resilience of the biosphere, establishing Green Microbiology as a conceptual and practical foundation for microbial sustainability science in a changing planet.

## 2 Chapter 2: From Sustainable Development to Planetary Resilience

The concept of sustainable development was first formally articulated in the Brundtland Commission's report in 1987 (World Commission on Environment and Development 1987). Nearly three decades later, in 2015, it was formally adopted and embedded in the United Nations' Sustainable Development Goals (SDGs) as a comprehensive framework for the alignment of environmental sustainability with social and economic growth and sustenance. Today, the concept of sustainable development is driven by the need to ensure that present‐day societies meet their current needs while preserving their future generations' ability to draw resources from the environment to meet their own needs. The current transition to sustainability is founded on poverty eradication efforts, promotion of universal health and quality education, climate action, protection of terrestrial and aquatic ecosystems, responsible consumption and production, and the provision of clean water and energy for all.

While previously sufficient, sustainable development no longer fully captures the depth of the environmental and ecological crises captured in Chapter 1. These compounding pressures call for the extension of the scope of the sustainability frontier beyond how to develop sustainably to how to strengthen the resilience of Earth systems so that they can absorb, adapt to, and recover from current and future challenges. This shift will require building the capacity of ecosystems, communities, and socio‐ecological networks to withstand disruptions, undergo adaptive transformations, and maintain the necessary production or resource distribution levels for current and future generations. This requires an audit of current production and consumption as well as the underlying ecological processes, including microbial processes.

The concept of Green Microbiology was previously framed as a microbial blueprint for sustainable development (Akinsemolu 2023). Its scope was primarily environmental sustainability, focusing on water and soil remediation, minimization of harmful chemical use, waste valorization, natural resource preservation and protection, ecosystem protection and preservation, and food sustainability through nutrient cycling and the production of biofertilizers, biopesticides, and animal feeds (Diaz‐Troya and Huertas 2024; Akinsemolu 2023). This perspective extends that framing toward planetary resilience by positioning microorganisms as foundational actors in ecological repair, climate adaptation, circular resource use and management, and microbial sustainability science. The earlier work established the seven principles of Green Microbiology, namely, sustainability, renewability, biodegradability, multifunctionality, biocompatibility, diversity, and collaborativity. This paper translates them into a research agenda that extends the scope of Green Microbiology beyond sustainable development to planetary resilience, exploring how microbial life supports the resilience of ecosystems, societies, and planetary systems.

## 3 Chapter 3: Defining Green Microbiology as a Framework

Green microbiology is an interdisciplinary framework that organizes and directs microbial science toward planetary resilience. It draws on the same scientific foundation as environmental microbiology, industrial microbiology, agricultural microbiology, and microbial biotechnology, but differs from each in purpose and organizing goal. While Green Microbiology does not constitute a fully developed scientific discipline yet, it is presented here as a framework with the potential to mature into an established scientific discipline with a distinct evaluative framework, a dedicated body of empirical research, and an institutional infrastructure. The conceptual architecture of the field of study is captured in Figure 1 below, demonstrating its pathway from the principles of sustainable development to its broader objective of planetary resilience.

**FIGURE 1:**
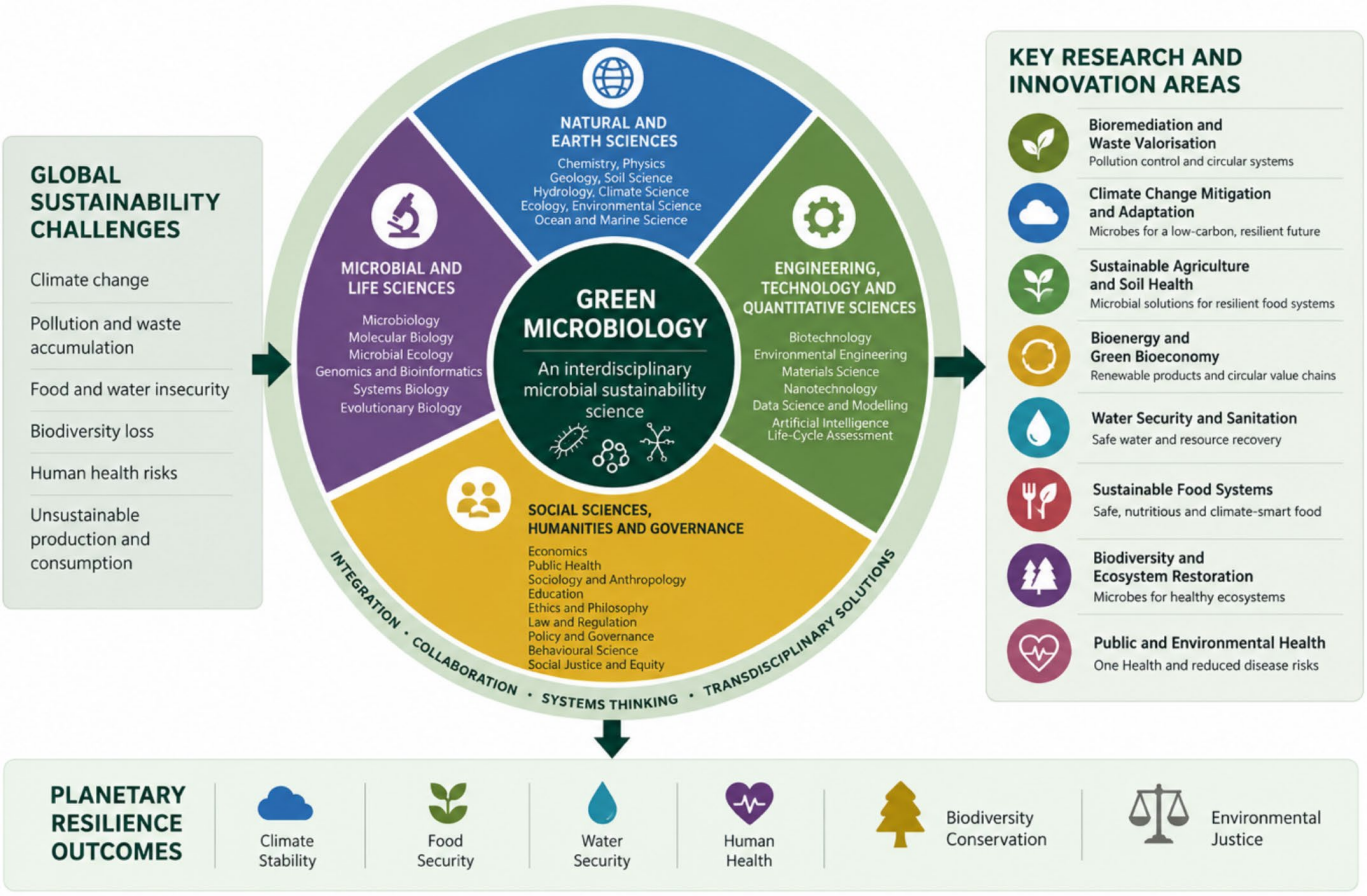
Conceptual framework of Green Microbiology for Planetary Resilience.

The distinction between Green Microbiology and environmental microbiology, industrial microbiology, agricultural microbiology, and microbial biotechnology lies in scope and orientation. Environmental microbiology studies microbes in the environment, exploring their diversity, ecological functions, and abundance in different environments. It is a substantially applied field, encompassing bioremediation, waterborne pathogen management, microbial risk assessment, and pollution control while advancing knowledge of microbial diversity and ecological function across terrestrial, aquatic, and atmospheric environments (Pepper et al. 2026). Although the two fields overlap substantially, Green Microbiology relies on the foundation laid by environmental microbiology to explore how microbial knowledge can be harnessed to sustain, restore, and transform human interactions with the natural world. It organizes microbial knowledge and applications across environmental, agricultural, industrial, and health‐related contexts, around a shared agenda of planetary health and resilience.

Industrial microbiology focuses on the industrial applications of microorganisms through the development and optimization of microbial processes for industrial, pharmaceutical, agricultural, and food applications, while agricultural microbiology explores microorganisms' interactions with the environment, soil, and plants (Trivedi et al. 2020; Nielsen and Keasling 2016). Comparatively, microbial biotechnology studies the technological applications of microorganisms in the production of natural bioactive compounds such as antibiotics, enzymes, organic acids, biofuels, and pharmaceuticals (Santos‐Beneit 2024). While its knowledge and applications are fundamental to Green Microbiology, microbial biotechnology is primarily productivity‐oriented and values yield, efficiency, and commercial value over sustainability. Green Microbiology, on the other hand, incorporates biotechnological approaches but views them from a sustainability lens, seeking to determine how microbial knowledge can help repair, sustain, and transform human‐Earth relationships.

As an emerging framework with the ambition and potential to mature into a recognized field, Green Microbiology requires a precise and authoritative definition that distinguishes it from existing disciplines, explains its scope and methods, and establishes its position within the broader architecture of sustainability science. This perspective proposes the following definition: Green Microbiology is the study and application of microorganisms, microbial communities, and microbial processes to advance sustainability, ecological restoration, climate resilience, circular resource use, environmental justice, and planetary health, while reducing the ecological footprint of microbiological science and biotechnology. This definition encompasses several dimensions that converge to give the field a distinctive identity. First, Green Microbiology is a science that is grounded in and supported by extensive research and knowledge in the scientific investigation of microbial physiology, ecology, genomics, and biogeochemistry. Second, it extends beyond generating knowledge to application. It seeks to translate microbial knowledge into practical interventions that address sustainability challenges. Third, it is value‐oriented, organizing microbial science around sustainability outcomes of justice, equity, and planetary health.

Green Microbiology shares its experimental tools and processes with existing subdisciplines of microbiology. As a discipline, its distinguishing feature is not a unique laboratory methodology but a distinct evaluative framework that assesses microbial research and technologies through sustainability outcomes, ecological impacts, equity considerations, and planetary resilience metrics. In practice, this framework asks whether a microbial intervention is ecologically safe for native microbial communities and the ecosystems they support, delivers a net environmental benefit across its full life cycle, strengthens a system's capacity to maintain function under stress, and has benefits that are distributed equitably within the communities where it is deployed and beyond. A mature Green Microbiology would also require its own institutional infrastructure, including dedicated scientific societies, journals, conferences, educational programmes, policy platforms, and research funding mechanisms explicitly organized around microbial sustainability science. Some building blocks for this infrastructure, such as the International Union of Microbiological Societies' call to action on microbes and planetary health (Rappuoli et al. 2023) and the IUCN Microbial Conservation Specialist Group (Gilbert et al. 2025), are already in place. A structure that connects them under a shared agenda is the missing link. Chapter 7 outlines how this link would be established over the next decade.

One of the defining properties of Green Microbiology is its inherent interdisciplinary nature, which establishes its main contribution alongside microbiological research. It draws from life sciences, including microbiology and ecology; physical and Earth sciences, including physics, chemistry, soil science and climate science; engineering and quantitative sciences; and social sciences, humanities and governance, including economics, public health, education, ethics, law and policy. It is connected to sustainability science through its focus on harnessing microbial processes for human, ecosystem, and overall planetary wellbeing. It is connected to One Health through its recognition that human, animal, plant, and environmental health are interconnected and mediated by shared microbial systems (Banerjee and van der Heijden 2023). It is connected to the circular bioeconomy through its focus on microbial‐mediated waste valorization and water and soil remediation. Finally, Green Microbiology is connected to environmental justice through its role in the protection and reclamation of ecosystems and its foundation on equality in the distribution of the benefits of microbial science. Ultimately, microbiologists are bound by the ethical responsibilities of transparency, equity, justice, and ecological accountability, which make up the core commitments of Green Microbiology. These connections and the field's commitments converge into six core pillars summarized in Table 1 below.

**TABLE 1:** Core pillars of green microbiology and their sustainability outcomes.

Pillar	Description	Sustainability outcome	References
Ecological Integration	In Green Microbiology, microbes are not isolated entities but inseparable components of broader ecological systems.	Biodiversity conservation Ecosystem health Sustainable land and water management.	Armstrong 2025; Zhou et al. 2024; Mishra et al. 2020
Circular Resource Use	Microbes close resource loops by converting domestic, industrial, and agricultural waste materials into valuable products	Reduced waste Promotion of circular bioeconomy Reuse of renewable materials	Jatoi et al. 2025; Rasool 2025
Climate Responsibility	Microbial sustainability science has solutions for greenhouse gas regulation, carbon cycling, and climate change responses	Climate change mitigation Carbon sequestration Methane removal	Dang et al. 2022; Kang et al. 2025; Orr et al. 2025; He et al. 2023
Green Laboratory Practice	Green laboratories are designed to reduce plastic waste, consume less energy and water, and reduce chemical waste	Reduced use of single‐use plastic Reduced environmental footprint Established blueprint for sustainable research practice	Tan et al. 2025; Freese et al. 2024
One Health Orientation	In Green Microbiology, human, animal, plant, and environmental microbiomes are considered and treated as interconnected systems	Integration of environmental and microbial health into global health, agriculture, and animal agricultural policy	Banerjee and van der Heijden 2023; Sessitsch et al. 2023; Flandroy et al. 2018; Soth et al. 2025
Responsible Innovation	The environmental impact of novel microbial technologies is evaluated	Prevention of ecological harm Established public trust Alignment of innovation with societal values	Cabrera‐Barjas et al. 2022; Taylor et al. 2023; Hassard et al. 2026

The novelty of Green Microbiology does not lie in the discovery of new microorganisms, methods, or biological processes but in establishing a mission‐oriented framework that reorganizes existing microbiological knowledge around planetary resilience as an explicit organizing principle. This reorganization has clear precedents. Notably, sustainability science emerged by integrating existing disciplines around sustainability challenges (Shrivastava et al. 2020). Green Microbiology adopts a similar approach, realigning microbiological research priorities, methodologies, and governance structures around climate resilience, biodiversity conservation, circular bioeconomy, environmental justice, and One Health objectives. It introduces three novel features. First, it introduces a criteria by which microbial research and technologies are evaluated, requiring established contributions to sustainability and a full life cycle assessment. Second, it holds microbiology research accountable for its environmental footprint. Finally, it treats equity, benefit‐sharing, and public participation as primary conditions. Together, these features establish Green Microbiology as a mission‐oriented framework that could mature into a new discipline.

## 4 Chapter 4: The Microbial Foundations of Planetary Resilience

Microbial resilience lays the foundation for planetary resilience through the five domains presented in Figure 2 below. Microorganisms make up the vast majority of Earth's biomass and genetic diversity, with at least one trillion microbial species populating the planet (Rappuoli et al. 2023). They are integral to the functioning of aquatic and terrestrial ecosystems and influence all other elements of the planet. Understanding all the specific ways in which microbes support, or undermine, planetary resilience is essential for driving the research agenda proposed in this Perspective.

**FIGURE 2:**
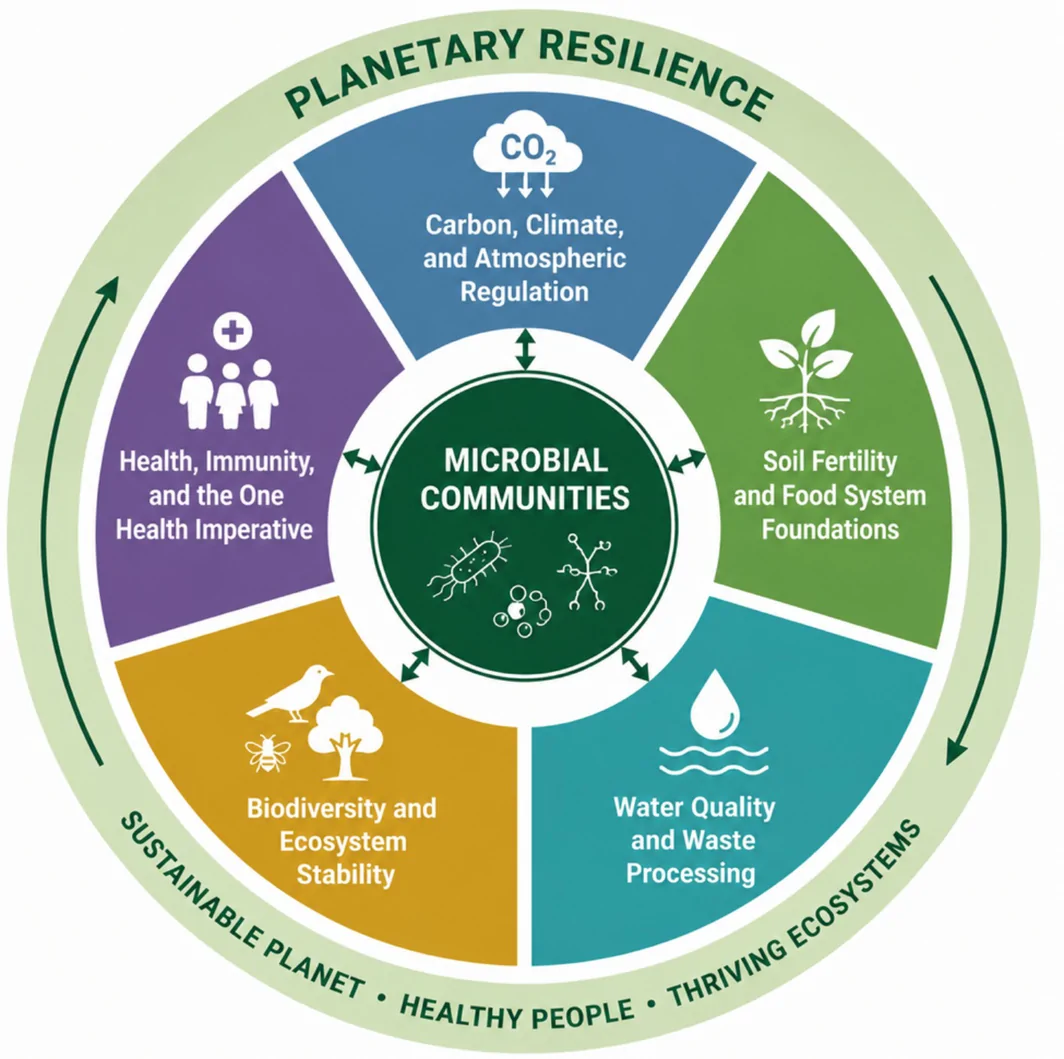
Planetary resilience framework showing how microbial communities support climate, soil, water, biodiversity, and health systems.

### 4.1 Carbon, Climate, and Atmospheric Regulation

Microorganisms are central to the carbon cycle. Bacteria and fungi in the soil decompose organic matter, mineralize carbon, and either store it in the soil in the form of minerals such as carbonates or release it into the atmosphere as carbon dioxide (Pradhan et al. 2025; Tao et al. 2023). Similarly, marine phytoplankton and cyanobacteria fix carbon dioxide through photosynthesis, acting as carbon sinks (Jia et al. 2022). In addition to carbon sequestration and decomposition of organic waste in the soil, microorganisms are crucial to the elimination of methane from the atmosphere. Methane‐producing microorganisms, which thrive in anaerobic environments such as rice paddies, swamps and wetlands, and domestic or industrial waste systems, decompose biomass into methane, which methanotrophs utilize as a source of carbon and energy, contributing to remediation (Bueno de Mesquita et al. 2023; Wen et al. 2017). Methanotrophic bacteria, in turn, oxidize the methane, creating a biological filter that substantially reduces the concentration of methane in the atmosphere (He et al. 2023). Notably, the contributions of microorganisms to long‐term carbon sequestration are an actively debated topic, with estimates of their capacity as carbon sinks varying across ecosystems, as translating laboratory‐scale volumes to field volumes remains a challenge. Soil degradation, wetland drainage, melting icebergs, ocean acidification, biodiversity loss, and any other disruptions of these microbial processes undermine the biosphere's self‐regulation capacity. Therefore, climate stability depends on the integrity of microbial communities.

### 4.2 Soil Fertility and Food System Foundations

Agricultural productivity relies monumentally on the metabolic activity of soil microorganisms. Nitrogen‐fixing bacteria convert atmospheric nitrogen into forms accessible to plants, such as nitrates, which are easily assimilated by plant roots and other soil microorganisms (Ladha et al. 2022). Biological Nitrogen Fixation (BNF) is the largest natural source of new nitrogen, reducing the need for synthetic nitrogen fertilizer (Reis Ely et al. 2025). Similarly, phosphate‐solubilizing bacteria and mycorrhizal fungi derive inorganic phosphorus from soil minerals and convert it into a bioavailable form, improving plant nutrient acquisition in phosphorus‐limited soils (Richardson and Simpson 2011). Soil‐borne decomposers, including actinomycetes, fungi, and various bacterial communities, mineralize crop residues and organic matter, releasing nutrients in bioavailable forms that are easily absorbed and used by plants. These microbial processes support and improve soil fertility sustainably compared to synthetic fertilizers and other agricultural chemicals. They are the cornerstones on which Climate‐smart agriculture, which seeks to produce food while reducing greenhouse gas emissions and building resilience to climate variability, is founded.

### 4.3 Water Security, Wastewater Treatment, and Bioremediation

This section addresses wastewater treatment, contaminant remediation, and decentralized water security solutions. The three microbial functions often occur together in practice but differ in their targets, operating settings, and technological maturity.

#### 4.3.1 Wastewater Treatment

Biological wastewater treatment plants rely on microbial consortia, including nitrifiers, denitrifiers, sulphate reducers, and polyphosphate‐accumulating organisms to remove organic matter, nitrogen, phosphorus, and pathogens from domestic, municipal, and industrial waste (Cydzik‐Kwiatkowska and Zielińska 2016). These processes combine predation and antagonism within microbial communities and adsorption of pathogens using biofilms and settling flocs with downstream physicochemical disinfection steps such as chlorination or UV treatment to eliminate pathogens from effluent. Municipal biological treatment for nutrient and organic matter removal is commercially mature and has been deployed widely (Cydzik‐Kwiatkowska and Zielińska 2016).

#### 4.3.2 Contaminant Remediation

Bioremediation processes harness the metabolic systems of microorganisms such as Pseudomonas, Dehalococcoides, Geobacter, and Rhodococcus species to either enzymatically degrade or co‐metabolize organic pollutants, pesticides, and hydrocarbons into less harmful byproducts. Heavy metals, on the other hand, are chemically altered and sequestered by species such as Geobacter through biosorption, bioaccumulation, or precipitation, reducing their mobility and bioavailability without eliminating them. Metal biosorption and bioaccumulation systems have reached pilot scale with a few commercial‐scale deployments in specific industrial and mining contexts, but broad industrial adoption remains limited (Lindholm‐Lehto 2019; Hidayatullah et al. 2026). Microbial microplastic degradation remains largely limited to laboratory research, with no concrete pathway to pilot‐scale studies or commercial deployment to date (da Silva et al. 2024).

#### 4.3.3 Decentralized Water Security

Microbial remediation systems are easily decentralized, offering cost‐effective, renewable, and non‐toxic water treatment solutions for communities and locations without access to centralized infrastructure. However, these are not universal properties of microbial remediation. Some of these applications depend on site conditions, contaminant load, and system design.

### 4.4 Biodiversity and Ecosystem Stability

Microorganisms are the most abundant and diverse living things on the planet. They enhance plant and soil resilience during adverse weather events, cycle nutrients in the soil in terrestrial ecosystems, degrade pollutants, and cycle nutrients in aquatic environments (Fierer 2017; Shi et al. 2021). Despite their fundamental roles in ecosystem stability, resilience against environmental disturbances, and renewability, these properties are not reflected in global diversity monitoring, conservation policy, and international environmental agreements. The unified research framework proposed here positions protecting microbial biodiversity as both an ecological imperative and a scientific priority.

### 4.5 Health, Immunity, and the One Health Imperative

One of the most significant research frontiers of modern biology is the relationship between microorganisms and human, animal, plant, and environmental health. One profound outcome of research into this relationship is the composition and magnitude of the human gut microbiome and its role in shaping the development of the immune system, regulating metabolic function, protecting human beings against enteric pathogens, regulating mood, memory, and cognition, and preventing inflammation (Kim et al. 2025; Cryan et al. 2019). Similarly, animal microbiomes influence livestock health, wildlife immunity, and the transmission of zoonotic diseases, while plant microbiomes contribute to crop resistance to drought, susceptibility to soil‐borne pathogens, and abiotic stress. Environmental microbiomes, on the other hand, which are found in the soil, water, and air, influence the human beings, plants, and animals that interact with them. One area of convergence of human, animal, and environmental microbiomes is Antimicrobial resistance, which results from antibiotic use in human medicine, veterinary practice, and agriculture, and spreads through these shared environmental reservoirs, posing a systemic threat to all three frontiers. Planetary resilience, therefore, requires a One Health approach to microbiome science by treating human, animal, plant, and environmental microbiomes as components of a single interconnected system.

## 5 Chapter 5: Research Frontiers in Green Microbiology

Green Microbiology is a promising transformative field of study with the capacity to drive planetary restoration, address the three planetary crises, and maintain overall planetary health. The translation of its collective frontiers from a conceptual framework into a productive scientific field requires their identification and conceptualization. This perspective has broken down Green Microbiology into eight frontiers representing the domains in which the field of study can generate the greatest value over the next 10 years. Since all eight frontiers are interconnected, individual progress in one domain will act as a catalyst for further advancement in the rest.

### 5.1 Frontier 1: Climate Microbiology

The role of microbial communities in climate regulation is only understood marginally at the mechanistic level. As a result, the potential contribution of microbial processes to climate change mitigation and climate adaptation is substantially underexploited (Cavicchioli et al. 2019). Key research areas to drive the discovery and accumulation of knowledge to this end include understanding the underlying mechanisms of soil microbiome in the stabilization and destabilization of soil organic matter amidst rising global temperatures and shifting precipitation patterns, the characterization of methanotrophic bacteria and their capacity for offsetting methane emissions, the capacity for microbial carbon mineralization and sequestration in degraded soil, and ways of advancing microbial electrosynthesis and carbon capture technology to convert organic waste and released CO_2_ into renewable energy (Tang et al. 2022; Liang et al. 2025; Peixoto et al. 2024; Zhu et al. 2022). Granted, microbial electrosynthesis is in its early stages of technological maturity, but laboratory studies show advanced readiness levels (Cabau‐Peinado et al. 2024). Beyond carbon capture technologies, climate microbiology can also contribute to ecosystem restoration. Research on the native microbial communities of mangroves, peatlands, wetlands, and degraded soils can support restoration strategies by identifying the microbial conditions under which these ecosystems recover their capacity to store carbon, which would help re‐establish long‐term carbon sinks while also supporting biodiversity conservation.

### 5.2 Frontier 2: Microbial Circular Bioeconomy

The concept of circular economy minimizes waste and maximizes the utility or value derived from a resource through its continuous use for as long as it remains productive. In the circular economy, waste is ideally eliminated through biological nutrient cycles that keep resources in circulation (Vural Gursel et al. 2022; Wolfaardt et al. 2025; Kirchherr et al. 2017; Nelson et al. 2026). This concept is founded on microbial metabolism. Microorganisms' natural processes can be adapted to convert crop biomass, food waste, organic waste, wastewater effluents, and industrial by‐products into biofuels, biogas, bioplastics, organic acids, amino acids, enzymes, single‐cell proteins, biofertilisers, and biodegradable packaging materials (Keasling et al. 2021). This can be achieved through consolidated bioprocessing approaches that rely on a single microbial platform that can simultaneously break down complex carbohydrates into simple sugars, ferment, and detoxify complex feedstocks, offering efficiency and sustainability advantages over multi‐stage chemical processes. Research priorities to advance this frontier and harness its applications include the engineering of robust mixed‐cultures capable of converting diverse waste streams, the development of microbial platforms that can degrade and allow the reuse of synthetic plastics, and the design of closed‐loop bioprocesses that reuse water and energy, and either reduce the need for chemical inputs or do away with synthetic inputs altogether.

### 5.3 Frontier 3: Food System Resilience

Microbial science presents various pathways across food supply chains for the achievement of resilient food systems. In primary production, biofertilizers, biopesticides, and other microbial disease and biological agents reduce the need for synthetic agricultural chemicals, bringing down the environmental cost of agriculture without compromising soil quality and fertility, food safety and quality, and the quantity of agricultural yield (Ferreyra‐Suarez et al. 2024; Ammar et al. 2023). Similarly, rhizosphere microbiome management approaches such as cover cropping and targeted inoculation, which reduce the need for tillage, improve nutrient uptake, and promote crop resilience, improve long‐term soil fertility, build resilience against extreme weather events, and protect the soil from erosion by reducing the need for mechanical disturbance (Trivedi et al. 2020; Amarasinghe et al. 2026). Beyond food production, fermentation enhances the nutritional value of food while extending its shelf life, creating flavour complexity, and reducing anti‐nutrients (Siddiqui et al. 2023; Marco et al. 2017). In animal farming, microorganisms are used in the production of alternative proteins derived from fungal mycelium and microalgae, lowering greenhouse emissions from livestock farming substantially.

### 5.4 Frontier 4: Water Security and Bioremediation

It is estimated that 71% of the global population faces water scarcity for a few months every year due to limited freshwater resources (Jaiswar et al. 2022). Microbial science is uniquely positioned to increase access to safe water, contribute to wastewater treatment, and support the remediation of pollutants from freshwater sources (Omohwovo 2024; Mustapha et al. 2026; Swapnil et al. 2023). Priority research areas in this frontier include the development of novel biological nutrient recovery systems that extract phosphorus and nitrogen from wastewater for use in organic fertilizers, the engineering of microbial fuel and electrolysis cells that can generate electrical energy or hydrogen during wastewater treatment, and the identification and cultivation of microbial consortia capable of degrading emerging contaminants including pharmaceuticals, personal care products, and microplastics, and the development of decentralized biosystems that can be constructed in wetlands, sewer systems, heavily polluted aquatic ecosystems, and regions with limited access to fresh water.

### 5.5 Frontier 5: Microbiomes and One Health

One Health is an integrated approach that recognizes the interconnectedness of human, animal, and environmental health (Danasekaran 2024; Lynggaard et al. 2025). It requires a robust microbiome science infrastructure guided by priority research areas such as metagenomic surveillance to identify species/strains, genes, and mutations associated with Antimicrobial Resistance (AMR), mobile genetic elements across the three domains of One Health in shared ecological contexts, and the relationship between longitudinal environmental microbiome diversity and the development and strengthening of human and animal immune systems. This is crucial to the assessment of non‐communicable disease risk, development of microbiome‐based therapies for infectious and inflammatory conditions, and the integration of environmental microbiome monitoring into public health surveillance systems, particularly early warning systems.

### 5.6 Frontier 6: Microbial Biodiversity Conservation

Microbial diversity and its conservation are some of the least studied subfields of Green Microbiology. As a frontier, microbial biodiversity conservation suffers secondary underdevelopment owing to the nature of microorganisms, which makes their diversity nearly impossible to evaluate using conventional macroecological methods (Vitorino and Bessa 2018; Mascarenhas et al. 2020). Consequently, it is difficult to determine the extent to which microbial diversity remains uncultivated and uncharacterised. Research priorities to address this gap include the development of standardized environmental DNA sampling and sequencing protocols to monitor global microbial biodiversity, the establishment of international archives of microbial genetic heritage, the inclusion of microbial diversity indicators in national biodiversity strategies and action plans under the Convention on Biological Diversity, and the commissioning of studies on the impact and consequences of microbial biodiversity loss in degraded ecosystems. This will provide the empirical basis for policy arguments in favour of microbial conservation.

### 5.7 Frontier 7: AI, Omics, and Predictive Microbial Sustainability Science

The convergence of metagenomics and other omics methodologies with machine learning will transform our capacity to understand, predict, and manage microbial systems at scale. Large‐scale metagenomic surveys such as the Earth Microbiome Project, the Global Ocean Survey, and national soil microbiome initiatives continuously generate foundational datasets that can be linked to environmental metadata and sustainability outcomes to support predictive modelling of microbial responses to climate change, land use, and pollution (Shaffer et al. 2022; Nesme et al. 2016; Tripathi and Nailwal 2020). Deep learning models trained on multi‐omic datasets can predict the capabilities and applications of uncultivated microorganisms, identify novel enzymes for biotechnological applications, and forecast how ecosystems would respond to different management scenarios. Synthetic biology platforms trained on genome editing data can simulate DNA synthesis and inform the design of microbial strains and communities for targeted sustainability applications. Research priorities to this end include the development of open‐access microbiome data platforms, the application of machine learning approaches trained on this data, and policy support for experimentation based on their output.

### 5.8 Frontier 8: Green Laboratory Practice

Microbiological and biological research has an invisible, yet significant contribution to the climate and waste crises. Ultra‐low‐temperature freezers, autoclaves, biosafety cabinets, and constant‐temperature incubators are energy‐intensive while single‐use plastics such as pipette tips, culture flasks, microcentrifuge tubes, and gloves generate large volumes of non‐recycled laboratory waste annually (Howes 2019; Freese et al. 2024; Alves et al. 2021). To reduce this adverse impact while maintaining the transformative work, research priorities in this frontier include life cycle assessments of standard microbiological procedures, the identification of substitutes, the development of reusable laboratory tools that are compatible with biosafety requirements, the design of energy‐efficient laboratory equipment, and the integration of sustainable procurement principles into institutional laboratory management. Finally, green laboratory principles should be embedded into undergraduate and postgraduate microbiology education, to ensure that the next generation of researchers approaches laboratory practice with environmental awareness.

## 6 Chapter 6: Risks, Ethics, Governance, and Justice

A scientifically credible and socially responsible framework for Green Microbiology must acknowledge the risks, ethical concerns, governance challenges, and risks of injustices and social inequalities that accompany the development and deployment of microbial technologies.

### 6.1 Biosafety and Biosecurity

Releasing microbial inoculants into the environment carries ecological risks that are not always anticipated beforehand. The introduced microorganisms may disrupt natural nutrient cycles, introduce microbes that outcompete native strains, spread diseases, or impair agricultural productivity (Li et al. 2024; Ladau et al. 2025). Microbial inoculants can also be weaponized to destroy food security and sovereignty, health, and ecosystems. This calls for robust risk assessment frameworks, extensive research and testing before the environmental deployment of microbial solutions, and strict regulation of microbial system engineering.

### 6.2 Justice, Equity, and Access

Microbial innovation and its benefits are not equitably distributed, and Green Microbiology could fall into existing patterns of inequality unless deliberately designed to be equitable. Research institutions and corporations in high‐income countries are often the owners of intellectual property such as information on microbial strains, biosynthetic gene clusters, and omics datasets, sometimes limiting access by researchers and communities in the Global South. Benefit‐sharing obligations under the Nagoya Protocol may not adequately guarantee access to researchers from marginalized communities even for marine and extremophile microorganisms collected from sovereign national territories. As a result, research priorities and funding flows in microbial science overwhelmingly reflect the interests of wealthy nations and their agricultural and pharmaceutical industries, sidelining less developed countries and regions. A just Green Microbiology must actively address these inequalities through capacity building in the Global South, open‐access technologies for low‐resource areas, and respecting indigenous and traditional knowledge associated with microbial resources.

### 6.3 Greenwashing and Technological Solutionism

Despite its name, Green Microbiology is not inherently green, and not every microbial product or process is genuinely sustainable. Biofuels derived from food crops compete for land usage with food production and may generate net carbon emissions. Bioplastics produced using microbes may not degrade as organically as presented, offering little environmental benefit. Claims of large‐scale carbon sequestration by soil microbiome may be exaggerated or wrongly idealized as they often lack rigorous long‐term field evidence. Therefore, Green Microbiology must insist on life cycle assessment and transparency.

### 6.4 Public Engagement and Democratic Governance

Finally, decisions about genetic modification, the release of inoculants into the environment, and large‐scale ecosystem interventions should not be made exclusively by scientists or regulatory bodies. They should require the participation of the public, communities in the targeted areas, and affected populations. Participatory governance mechanisms should be established for every group with legitimate interests in how, when, and where microbial technologies are designed, deployed, and governed.

## 7 Chapter 7: A 2026–2036 Research Agenda

This perspective proposes 10 priority areas for the decade 2026 to 2036 to drive the translation of Green Microbiology from a framework into an institutionalized field of science, education, and policy. The agenda is intentionally interdisciplinary: rather than treating microbial research as an independent pathway to planetary resilience, it positions microbial science within integrated collaborations spanning the natural and Earth sciences, engineering and technology, social sciences, humanities, economics, education, law, public policy, and community knowledge. Across the 10 priorities, microbiology provides a foundational understanding of microbial systems, while complementary disciplines contribute the knowledge, methods, technologies, governance mechanisms, and implementation pathways required to address complex planetary challenges. Designed to be actionable, measurable, and interconnected, these priorities are presented in Table 2 and elaborated below.

**TABLE 2:** Green microbiology 2026–2036: An interdisciplinary research and policy agenda.

Numbers	Priority area	Interdisciplinary integration	Key research actions	2036 milestones
1	Define Global Standards	Microbiology; sustainability science; ecology; life‐cycle assessment; economics; ethics; law; policy	Develop criteria for evaluating environmental impact Establish collaboration between scientific societies, learning institutions, and intergovernmental bodies	A published, recognized, and adopted assessment framework for microbial sustainability claims.
2	Microbial Climate Solutions	Microbiology; biogeochemistry; soil science; climate science; ecology; agronomy; environmental economics; policy	Fund, support, and incentivize dedicated research on soil carbon sequestration and methane removal by combining microbiology with biogeochemistry and soil science Partner with agronomists, ecologists, farmers, and environmental economists to support climate‐smart agriculture and ecosystem restoration programs	Sustained funding for climate microbiology research.
3	Advancing Microbial‐Driven Circular Bioeconomy	Microbiology; chemical and biochemical engineering; materials science; industrial ecology; economics; life‐cycle assessment	Establish partnerships with chemical engineers and material scientists to support the transition of microbial conversion of organic waste, wastewater, agricultural residues, plastics, and industrial by‐products from laboratory to industrial applications	Pilot‐scale validation and industrial adoption of promising waste‐to‐value processes mediated by microorganisms.
4	One Health	Microbiology; medicine; veterinary science; public health; epidemiology; ecology; environmental health; policy	Integrate environmental microbiomes into One Health frameworks in collaboration with veterinary and public health scientists and epidemiologists Establish coordinated global AMR microbiome surveillance that integrate public health policy	Formal inclusion of environmental microbiome data in The Quadripartite Joint Secretariat on AMR reports.
5	Protecting Microbial Biodiversity	Microbiology; conservation biology; ecology; environmental law; policy science; biodiversity governance	Collaborate with conservation biologists, international environmental lawyers, and policy scientists to expand international environmental governance to include microbial diversity targets Integrating microbial diversity into conservation frameworks and biodiversity monitoring	Development of tracked indicators for microbial diversity.
6	Developing Microbial Technologies for the Global South	Microbiology; engineering; agriculture; public health; economics; development studies; community knowledge	Invest in research on affordable, decentralized water filtration and treatment biosystems Developing nitrogen fixation inoculants for low‐resource agricultural settings in collaboration with agronomists and farmers Scaling microbial food preservation and household energy systems	The availability of low‐cost tools such as microbial water filters or nitrogen‐fixing in underserved communities.
7	Ethical Microbial Governance	Microbiology; ethics; law; political science; policy; sociology; risk science; public engagement	Establishing participatory governance frameworks for synthetic biology, biosafety governance frameworks for environmental microbial releases, and ethics review processes for synthetic biology applications Developing benefit‐sharing agreements for microbial resources Establishing benefit‐sharing frameworks for microbial genetic resources Establishing public engagement mechanisms for controversial technology decisions	The global adoption of a benefit‐sharing framework for microbial resources.
8	Green Lab Transformation	Microbiology; sustainability science; engineering; education; behavioural science; institutional policy	Mandating life cycle assessments for laboratory operations Conducting energy audits and setting reduction targets for research buildings Implementing sustainable procurement policies and waste reduction mandates Integrating laboratory sustainability into academic curricula and research culture	Integration of sustainability standards into microbiology and scientific laboratories worldwide.
9	Responsible AI in Microbial Science	Microbiology; AI; data science; statistics; software engineering; ethics; data governance	Development of open and interoperable microbiome data platforms in collaboration with data scientists and software engineers Creation of AI governance standards for predictive microbial science Equitable participation in microbiome data generation and use	Microbiome platforms adopt the existing MIxS data standard to allow the reliable comparison of results across labs and studies.
10	Institutionalization of Green Microbiology	Microbiology; education; sustainability science; social sciences; policy; law; science communication	Establishment of dedicated Green Microbiology scientific societies, journals, and conferences Development of textbooks and joint curricula for the field in collaboration with educators and scientists from interconnected disciplines Creation of policy engagement platforms to connect microbial science with global sustainability governance	The establishment of a dedicated Green Microbiology scientific society, journal, and conference.

### 7.1 Define Global Standards for Green Microbiology

Currently, there are no widely accepted criteria for determining whether a microbial technology or research programme qualifies as genuinely green. Scientific societies, learning institutions, and intergovernmental bodies should collaborate to produce a Green Microbiology assessment framework grounded in life cycle thinking, ecological safety, social equity, and long‐term sustainability. The International Union of Microbiological Societies (IUMS) has already issued a call to action on microbes and planetary health, providing a forum bringing together the societies and researchers who would need to be involved in developing a standard framework for Green Microbiology (Rappuoli et al. 2023).

### 7.2 Build Microbial Solutions for Climate Resilience

A coordinated international research program on climate microbiology is imperative. It should have dedicated funding for microbial research on soil carbon, methane mitigation, climate‐smart agriculture, ecosystem restoration, and carbon‐negative biotechnologies. It should support data sharing, have an open‐access publication, and include Global South participation as a binding commitment.

### 7.3 Advance Microbial Circular Economy Systems

The microbial conversion of organic waste, wastewater, agricultural residues, plastics, and industrial by‐products into energy, biomaterials, and nutrients should be scaled through coordinated investment and open‐access bioprocess protocols.

### 7.4 Integrate Microbiomes Into One Health Policy

Environmental microbiomes should be recognized as essential components of the global health infrastructure, microbial diversity indicators integrated into One Health monitoring frameworks, and global AMR microbiome surveillance established. This can build on existing infrastructure established by the One Health Quadripartite (FAO, UNEP, WHO, and WOAH).

### 7.5 Protect Microbial Biodiversity

International environmental governance should be expanded to include microbial diversity targets, which should be integrated into conservation frameworks, biodiversity monitoring, and ecosystem restoration planning. The groundwork for this frontier has begun through the launch of a Microbial Conservation Specialist Group in 2025 by the IUCN, which became the first global safeguard mechanism for microbial biodiversity (Gilbert et al. 2025).

### 7.6 Develop Microbial Technologies for the Global South

Many of the populations most severely affected by soil degradation, water insecurity, food insecurity, and prevalent infectious disease outbreaks are located in low‐ and middle‐income countries that have limited access to advanced microbial technologies. Investment in affordable, decentralized microbial solutions, particularly water filters, nitrogen fixation inoculants, fermentation‐based food preservation microbial communities, and biogas for household energy production, can deliver significant sustainability benefits at modest cost.

### 7.7 Create Ethical Governance for Microbial Innovation

This includes biosafety governance for environmental microbial releases, benefit‐sharing frameworks for microbial genetic resources, ethics review processes for synthetic biology applications, and public engagement mechanisms for controversial or material decisions.

### 7.8 Establish Green Microbiology Laboratories and Education

Transforming green laboratory practice requires energy audits and reduction targets for research buildings, sustainable procurement policies, waste reduction mandates, and the integration of laboratory sustainability into academic curricula and research culture. Compared to the other priorities, existing infrastructure for this frontier is significantly mature, with My Green Lab, a global nonprofit certification body recognized by the UN Race to Zero campaign, already helping scientific laboratories reduce their environmental impact by providing certification against sustainability criteria, and the International Microbiology Literacy Initiative (IMiLI) providing freely available microbiology education resources globally (Timmis et al. 2019). This priority's task is to leverage these resources to expand content, resources, and sustainability criteria specific to Green Microbiology.

### 7.9 Use AI Responsibly in Microbial Sustainability Science

The responsible deployment of AI in microbial sustainability science requires the development of open, transparent, and reproducible data platforms that enable equitable participation in the generation and use of microbiome data.

### 7.10 Build Green Microbiology Institutions

Finally, dedicated scientific societies, journals, conferences, textbooks, curricula, and policy engagement platforms are necessary to transform Green Microbiology into a functioning field. These institutions will establish the institutional architecture necessary to support a global community of practice in microbial sustainability science.

## 8 Conclusion

Microorganisms are among the smallest forms of life but have a significant impact on planetary health. They form the invisible infrastructure of the biosphere upon which the foundation for planetary restoration lies. Green Microbiology offers a novel framework for the reorganization of existing knowledge of microbial communities and processes to advance planetary resilience. Its distinctive contribution lies not in creating a separate set of microbiological methods, but in integrating microbial science with natural and Earth sciences, engineering and technology, social sciences, humanities, economics, education, law, and policy to address complex planetary challenges. The research agenda proposed here calls for international and interdisciplinary collaboration, sustained investment in advancing knowledge, and genuine commitment to justice and equity. It requires microbiologists to engage with one another and with climate scientists, ecologists, social scientists, engineers, policymakers, farmers, health workers, and communities to acknowledge and leverage microbial sustainability science as a scientific imperative on which the resilience of Earth systems is founded. In this sense, Green Microbiology positions interdisciplinary integration as a core mechanism for translating microbial knowledge into sustainable and equitable outcomes. The coming decade will be critical for planetary restoration. Decisions made between now and 2036 about how we manage our soils, water, food, waste, and energy will have consequences that will be felt for centuries. Green Microbiology invites science to look again at the invisible majority of life and recognize microorganisms not only as agents of disease or tools of industry, but as essential partners in the repair, resilience, and renewal of a changing planet.

## Author Contributions

**Adenike A. Akinsemolu:** conceptualization, writing – original draft, writing – review and editing, validation, supervision. **Helen Onyeaka:** writing – original draft, writing – review and editing, supervision, validation.

## Funding

The authors have nothing to report.

## Conflicts of Interest

The authors declare no conflicts of interest.

## Data Availability

The authors have nothing to report.
